# The age-related impact of surviving sarcoma on health-related quality of life: data from the SURVSARC study

**DOI:** 10.1016/j.esmoop.2021.100047

**Published:** 2021-01-27

**Authors:** C. Drabbe, W.T.A. Van der Graaf, B.H. De Rooij, D.J. Grünhagen, V.L.M.N. Soomers, M.A.J. Van de Sande, L.B. Been, K.B.M.I. Keymeulen, I.C.M. van der Geest, W.J. Van Houdt, O. Husson

**Affiliations:** 1 Department of Medical Oncology, Netherlands Cancer Institute, Amsterdam, The Netherlands; 2 Department of Medical Oncology, Erasmus MC Cancer Institute, Rotterdam, The Netherlands; 3 Netherlands Comprehensive Cancer Organization, Utrecht, The Netherlands; 4 Department of Medical and Clinical Psychology, Tilburg University, Tilburg, The Netherlands; 5 Department of Surgical Oncology, Erasmus MC Cancer Institute, Rotterdam, The Netherlands; 6 Department of Medical Oncology, Radboud University Medical Centre, Nijmegen, The Netherlands; 7 Department of Orthopaedic Surgery, Leiden University Medical Centre, Leiden, The Netherlands; 8 Department of Surgical Oncology, University Medical Centre Groningen, Groningen, The Netherlands; 9 Department of Surgical Oncology, Maastricht University Medical Centre, Maastricht, The Netherlands; 10 Department of Orthopaedics, Radboud University Medical Centre, Nijmegen, The Netherlands; 11 Department of Surgical Oncology, Netherlands Cancer Institute, Amsterdam, The Netherlands; 12 Institute of Cancer Research, Sutton, London, UK

**Keywords:** sarcoma, health-related quality of life, survivorship, age-related, adolescents and young adults

## Abstract

**Background:**

Health-related quality of life (HRQoL) data of sarcoma survivors are scarce and the impact of age remains unclear. The aims of this population-based study were to (i) compare HRQoL scores amongst three age-groups [adolescents and young adults (AYA, aged 18-39 years), older adults (OA, aged 40-69 years) and elderly (aged ≥70 years)]; (ii) compare HRQoL of each sarcoma survivor age group with an age- and sex-matched normative population sample; (iii) determine factors associated with low HRQoL per age group.

**Methods:**

Dutch sarcoma survivors, who were 2-10 years after diagnosis, were invited to complete the European Organization for Research and Treatment of Cancer Quality of Life Questionnaire-Core 30-questions questionnaire on HRQoL.

**Results:**

In total, 1099 survivors (58% response rate) completed the questionnaire: 186 AYAs, 748 OAs and 165 elderly. The median time since diagnosis for all patients was 5.2 years. Bone sarcomas were seen in 41% of AYAs, 22% of OAs and in 16% of elderly survivors (*P* < 0.01). AYA and OA survivors reported statistically significant and clinically meaningful worse physical, role, cognitive, emotional and social functioning compared with a matched norm population, which was not the case for elderly survivors. AYAs reported significantly worse scores on emotional and cognitive functioning compared with OA and elderly survivors. Malignant peripheral nerve sheath tumour, osteosarcoma and chordoma were the subtypes of which survivors reported the lowest HRQoL scores in comparison with the norm. For all age groups, chemotherapy, having a bone sarcoma and having comorbidities were most frequently associated with low scores on HRQoL subscales, whereas a shorter time since diagnosis was not.

**Conclusion:**

In this nationwide sarcoma survivorship study, the disease and its treatment had relatively more impact on the HRQoL of AYA and OA survivors than on elderly survivors. These results emphasise the need for personalised follow-up care that not only includes risk-adjusted care related to disease relapse, but also age-adjusted care.

## Highlights

• In the very heterogeneous sarcoma survivor population, there is also a wide variety in HRQoL scores.
• Sarcoma and its treatment have a greater impact on the HRQoL of younger survivors than on the HRQoL of older survivors.
• Younger survivors reported worse scores on emotional and cognitive functioning compared with older survivors.
• Receiving chemotherapy, having a bone sarcoma and comorbidities were most frequently associated with low HRQoL scores.
• A shorter time since diagnosis was not associated with low HRQoL scores.

## Introduction

Sarcomas are rare tumours originating from mesenchymal stem cells and account for approximately 1% of adult malignancies. With more than 70 histological subtypes, sarcomas are extremely heterogenous tumours that affect people of all ages and can occur at any anatomical site.^1^ Broadly, a histological distinction can be made between soft tissue sarcomas (STS) and bone sarcomas (BS), with a 5-year survival rate of 55%-60% and 50%-55%, respectively.^2^, ^3^, ^4^ These poor outcomes are at least partially ascribed to delay in diagnosis, advanced disease at presentation, biological aggressiveness and, due to its heterogeneity, a relative lack of clinical trials.^1^^,^^5^, ^6^, ^7^, ^8^, ^9^

Sarcomas may present with complex symptoms and the clinical picture can vary from indolent to a highly aggressive phenotype.^1^ The latter consequentially leads to a high burden of symptoms and requires intensive treatment, potentially resulting in long-term side-effects and disabilities in survivors,^10^, ^11^, ^12^ which in turn can result in a decreased health-related quality of life (HRQoL).^13^ HRQoL is a patient reported outcome and encompasses physical, social, psychological, cognitive and spiritual aspects of well-being. Both HRQoL and survivorship are increasingly considered important components of patient-centred care and research suggests that cancer patients consider HRQoL an important treatment outcome.^14^, ^15^, ^16^

In personalising the care of sarcoma patients, much attention is paid to the various histological subtypes, their treatment and involved specialists. However, less attention has been paid to age aspects that are linked to sarcoma, even though sarcomas demonstrate an extremely diverse nature across the age spectrum both in terms of subtypes and incidence.^17^ In addition, the challenges that cancer patients in general face vary greatly at different ages. Adolescent and young adult (AYA) sarcoma patients are diagnosed at an emotionally, cognitively and socially challenging time in their lives, which may interfere with the acquisition of regular developmental milestones.^18^ In contrast, key issues that must be considered in treatment of elderly patients are physiological changes associated with aging, functional and role functioning, comorbidities, cognitive status and polypharmacy.^19^ Lack of representation in clinical trials is seen in both AYA and elderly patients.^20^^,^^21^ A recent study identified significant age-related differences in the sarcoma patient journey that are not only related to variations in sarcoma subtypes, such as more incorrect diagnoses and higher burden of treatment in younger patients and less referral to rehabilitation services in the elderly.^22^

HRQoL in sarcoma survivors has been marginally researched. This is a nationwide study with the purpose of assessing HRQoL in sarcoma survivors. The aim of this study is to (i) assess age-related differences in HRQoL between AYA (aged 18-39 years), older adult (OA, aged 40-69 years) and elderly (aged ≥70 years) sarcoma survivors. In addition, (ii) general HRQoL will be compared between sarcoma survivors and an age- and sex-matched normative sample and (iii) patient, tumour and treatment characteristics associated with low HRQoL score will be determined.

## Methods

### Study design and participants

This SURVSARC study is an exploratory population-based cross-sectional questionnaire study among adult (≥18 years of age) sarcoma survivors, registered in the Netherlands Cancer Registry (NCR). Sarcoma survivors diagnosed between 1 January 2008 and 31 December 2016 at one of the six participating sarcoma expertise centres (Radboud University Medical Centre, Antoni van Leeuwenhoek/The Netherlands Cancer Institute, University Medical Centre Groningen, Leiden University Medical Centre, Erasmus Medical Centre Rotterdam, Maastricht University Medical Centre) were eligible. Exclusion criteria were cognitive impairment and physical condition too poor to participate. Survivors with desmoid fibromatosis, grade I chondrosarcoma, gastrointestinal stromal tumours, atypical lipomatous tumours or giant-cell tumours were also excluded considering the indolent clinical behaviour and less aggressive treatment strategies. Ethical approval was obtained from the medical ethical committee of the Radboud University Medical Centre (2017-3944) and the study was registered in the Dutch Trial Registry (NTR-7253).

### Data collection

Eligible sarcoma survivors were invited by their (former) treating physician and if informed consent was obtained, participants were able to complete the questionnaire. The NCR contains data on patient, tumour and treatment characteristics of all newly diagnosed cancer patients in the Netherlands. Demographic, clinical and treatment characteristics were obtained from this database. All histology was verified through electronic patient records. Completion of the questionnaire was conducted between October 2018 and June 2019 within the Patient Reported Outcomes Following Initial treatment and Long-term Evaluation of Survivorship (PROFILES) data management system; patients were therefore 2-10 years after diagnosis.^23^

### Study measures

Marital status, educational level and employment status were self-reported by the participants. Treatment characteristics were patient-reported and any missing data were supplemented with treatment data from the NCR. In order to report on the age-related HRQoL in sarcoma survivors, participants were divided into three age categories according to their age at diagnosis; adults and young adolescents (aged 18-39 years), OAs (aged 40-69 years) and elderly survivors (aged ≥70 years).

### HRQoL

In order to assess cancer-generic HRQoL, sarcoma survivors completed the Dutch version of the European Organization for Research and Treatment of Cancer Quality of Life Questionnaire-Core 30-questions (EORTC-QLQ-C30).^24^ It was designed for cancer patients in general and consists of 30 items. The questionnaire contains five functional scales, a global QoL scale, three symptom scales and a number of single symptom items. A linear transformation of all scales and single item measures was conducted and scores ranged from 0 to 100.

### Normative sample

An age-matched and sex-matched normative sample without cancer was obtained from CentERdata, a research institute at Tilburg University, using a household panel representative of the population in the Netherlands. The panel members were randomly matched to each age group of sarcoma survivors separately based on sex and age at the time of questionnaire completion.^25^ A total of 186 panel members were matched to the 186 AYA survivors (ratio 1 : 1), 901 panel members were matched to the 748 OA survivors (ratio 1 : 1.2) and 232 panel members were matched to the 165 elderly survivors (ratio 1 : 1.4).

### Statistical analyses

An anonymous comparative analysis between responders and non-responders was conducted by an NCR employee and non-responder data were not shared with the research team. When comparing groups, chi-square tests were used for categorical variables, whereas independent samples *t*-tests and one-way analysis of variance were used for continuous variables.

Analysis of covariance (ANCOVA) was conducted to compare HRQoL functioning scales and symptom scales between survivors and the normative population by age group. These analyses were corrected only for age at the time of questionnaire completion and not for other potential confounding factors (e.g. education, partner, comorbidity) since variance between survivors and the norm could be caused by having cancer and the subsequent treatment. In addition to the statistical significance, the clinical relevance of the difference in scores was determined according to the Evidence-Based Guidelines for Determination of Sample Size and Interpretation of EORTC QLQ-C30 as determined by Cocks et al.^26^ ANCOVA was conducted to compare the HRQoL between the three age groups, correcting for time since diagnosis, comorbidity and whether patients had received chemotherapy. In case of significant effects, Bonferroni *post hoc* tests were carried out.

Univariate, age-stratified logistic regression analyses were used to determine the association between patient, tumour and treatment characteristics and clinically relevant low scores on functioning and symptom scales.

All statistical analyses were carried out using SPSS Statistics (IBM Corporation, version 26.0, Armonk, NY) and *P* values < 0.05 were considered statistically significant.

## Results

### Responders versus non-responders

In total, 1887 sarcoma survivors were approached, of whom 1099 provided informed consent and completed the questionnaire. The response rate for AYAs was 41%, for OAs 66% and for elderly 53%. Elderly responders were more often male than non-responders (*P* < 0.01) and AYA and OA responders were older than non-responders, whereas elderly responders were younger than non-responders (*P* < 0.01). No statistically significant differences were observed for STS versus BS between responders and non-responders, stratified by age group.

### Sociodemographic and clinical characteristics

Patient, tumour and treatment characteristics of all 1099 responding sarcoma survivors are reported in Table 1. Based on their age at diagnosis, 186 (17%) were AYAs (18-39 years), 748 (68%) OAs (40-69 years) and 165 (15%) were elderly (≥70 years) survivors. The median time since diagnosis for all survivors was 5.2 years (range 1.7-11.3). Some 45% of AYA survivors, 54% of OAs and 66% of elderly survivors were male (*P* < 0.01). Amongst AYAs, 78% had a partner, 83% of OAs had a partner and 68% of elderly survivors had a partner (*P* < 0.01). AYAs were most often highly educated (52%), followed by OAs (37%) and elderly (21%) age groups (*P* < 0.01). Relatively seen, BS were most often diagnosed in AYAs (41%), then in OAs (22%) and least often in elderly survivors (16%).

**Table 1:** Overall patient, tumour and treatment characteristics stratified by age group

	Total *N* = 1099	AYA (age 18-39 years) *N* = 186	OA (age 40-69 years) *N* = 748	Elderly (age ≥70 years) *N* = 165	*P* value
Sex					0.001
Male	596 (54.2)	84 (45.2)	404 (54.0)	108 (65.5)	
Female	503 (45.8)	102 (54.8)	344 (46.0)	57 (34.5)	
Age at diagnosis (years), median (range) Age at questionnaire (years), median (range)	56 (18-90) 62 (21-94)	30 (18-39) 37 (21-49)	57 (40-69) 62 (42-79)	75 (70-90) 80 (73-93)	
Time since diagnosis in months					<0.001^a^
Mean (±SD)	67.4 (30.4)	75.7 (31.1)	67.2 (30.5)	58.8 (30.4)	
Comorbidities					<0.001
0	369 (33.6)	110 (59.1)	240 (32.1)	19 (11.5)	
1	355 (32.3)	59 (31.7)	235 (31.4)	61 (37.0)	
≥2	375 (34.1)	17 (9.2)	273 (36.5)	85 (51.5)	
Histology					STS versus BS
STS	829 (75.4)	110 (59.1)	581 (77.7)	138 (83.6)	<0.001
Dermatofibrosarcoma protuberans	74 (6.7)	24 (12.9)	47 (6.3)	3 (1.8)	
Liposarcoma	177 (16.1)	25 (13.4)	128 (17.1)	24 (14.5)	
Myxofibrosarcoma	137 (12.5)	4 (2.2)	96 (12.8)	37 (22.4)	
Leiomyosarcoma	113 (10.3)	9 (4.8)	87 (11.6)	17 (10.3)	
Rhabdomyosarcoma	15 (1.4)	6 (3.2)	9 (1.2)	0 (0.0)	Histological subtypes
MPNST	34 (3.1)	11 (5.9)	20 (2.7)	3 (1.8)	<0.001^b^
Synovial sarcoma	35 (3.2)	10 (5.4)	24 (3.2)	1 (0.6)	
Vascular sarcoma	43 (3.9)	2 (1.1)	30 (4.0)	11 (6.7)	
Other STS	201 (18.3)	19 (10.2)	140 (18.7)	42 (25.5)	
BS	270 (24.6)	76 (40.9)	167 (22.3)	27 (16.4)	
Osteosarcoma	69 (6.3)	29 (15.6)	34 (4.5)	6 (3.6)	
Chondrosarcoma	130 (11.8)	26 (14.0)	89 (11.9)	15 (9.1)	
Chordoma	30 (2.7)	1 (0.5)	23 (3.1)	6 (3.6)	
Ewing sarcoma	28 (2.5)	16 (8.6)	12 (1.6)	0 (0.0)	
Other BS	13 (1.2)	4 (2.2)	9 (1.2)	0 (0.0)	
Clinical staging					0.002
Stage I	411 (44.8)	70 (46.1)	281 (45.0)	60 (42.3)	
Stage II	315 (34.3)	54 (35.5)	208 (33.3)	53 (37.3)	
Stage III	134 (14.6)	10 (6.6)	98 (15.8)	26 (18.3)	
Stage IV	58 (6.3)	18 (11.8)	37 (5.9)	3 (2.1)	
Missing	181	34	124	23	
Grade					0.209
Low grade	542 (57.1)	83 (53.5)	382 (59.0)	77 (52.4)	
High grade	407 (42.9)	72 (46.5)	265 (41.0)	70 (47.6)	
Missing	150	31	101	18	
Localisation					0.145
Head and neck	72 (6.6)	15 (8.1)	48 (6.4)	9 (5.5)	
Thoracic/breast	107 (9.7)	20 (10.8)	67 (9.0)	20 (12.1)	
Abdominal	133 (12.1)	11 (5.9)	102 (13.6)	20 (12.1)	
Skin	109 (9.9)	25 (13.4)	64 (8.6)	20 (12.1)	
Pelvis	84 (7.6)	12 (6.5)	59 (7.9)	13 (7.9)	
Upper extremities	116 (10.6)	19 (10.2)	76 (10.2)	21 (12.7)	
Lower extremities	405 (36.9)	67 (36.0)	286 (38.2)	52 (31.5)	
Other	73 (6.6)	17 (9.1)	46 (6.1)	10 (6.1)	
Treatment					<0.001^b^
Surgery only	464 (42.3)	73 (39.2)	315 (42.1)	76 (46.9)	
RT only	16 (1.5)	3 (1.6)	10 (1.3)	3 (1.9)	
CT only	8 (0.7)	1 (0.5)	7 (0.9)	0 (0.0)	
Surgery & RT	430 (39.3)	48 (25.8)	308 (41.3)	74 (45.6)	
Surgery & CT	79 (7.2)	36 (19.4)	40 (5.3)	3 (1.9)	
RT & CT	11 (1.0)	5 (2.7)	6 (0.8)	0 (0.0)	
Surgery & RT & CT	88 (8.0)	20 (10.8)	62 (8.3)	6 (3.7)	
Missing	3	0	0	3	

AYA, adolescents and young adults; BS, bone sarcoma; CT, chemotherapy; MPNST, malignant peripheral nerve sheath tumour; OA, older adults; RT, radiotherapy; SD, standard deviation; STS, soft tissue sarcoma.

a Bonferroni post hoc test corresponds to statistically significance of one-way ANOVA.

b Fisher’s exact (2-sided, Monte Carlo simulation).

### Sociodemographic characteristics of survivors and the normative sample

An age-stratified comparative analysis of all 1099 responding sarcoma survivors compared with the 1319 individuals from the normative sample was conducted. Due to the younger age of the normative population compared with the study participants, the age matching based on age categories for the elderly population was imperfect. The age at the time of the survey was significantly higher in elderly survivors compared with their norm (*P* < 0.01). OA survivors had a partner more frequently than the OA norm (*P* < 0.01). Compared with elderly sarcoma survivors, individuals in the elderly norm were more often highly educated (*P* < 0.01). Both the AYA and elderly norm were more frequently reported to have either no comorbidities or two or more comorbidities compared with the survivors in their corresponding age groups.

### Comparison of HRQoL between survivors and the norm according to age

**Figure 1:**
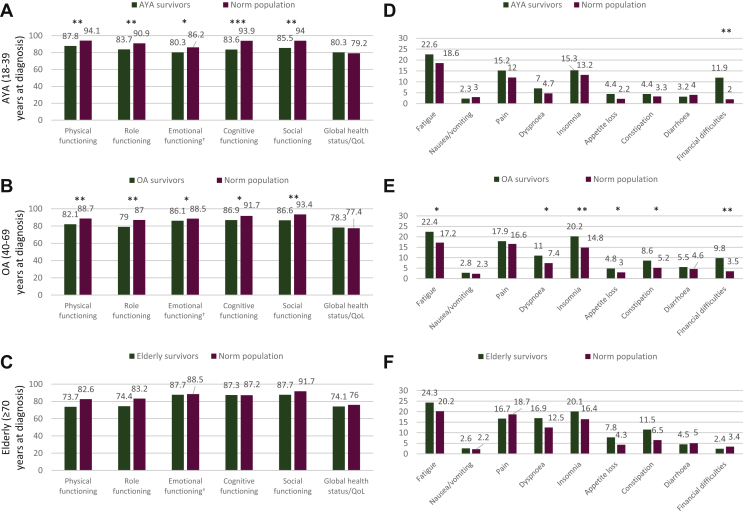
Differences in HRQoL functioning scales and symptom scores between sarcoma survivors and an age- and gender-matched normative population, stratified by age group. All analyses were corrected for age at time of filling out the questionnaire. Age groups were established using the age at diagnosis. (A and D) AYA, (B and E) OA, (C and F) elderly. AYA, adolescents and young adults; OA, older adults; QoL, quality of life. ^a^The clinical relevance of the emotional functioning score was determined according to the role functioning interpretation guideline. ∗*P* < 0.05 and trivial effect. ∗∗*P* < 0.01 and small effect. ∗∗∗*P* < 0.01 and medium effect.

In comparison with an age- and sex-matched normative sample, both AYA and OA sarcoma survivors reported significantly lower scores on five of the six functional scales (Figure 1A and B). In the elderly, no functional scales differed significantly between survivors and the norm after correction for age was applied (Figure 1C).

On the nine symptom scores, AYA survivors only reported significantly worse scores on financial difficulties which were of small clinical importance (Figure 1D). In OA, six symptom scores were significantly worse for survivors compared with their norm (Figure 1E). Within the elderly age group, survivors reported no statistically significant higher scores in comparison with the elderly norm population (Figure 1F).

### Comparison of HRQoL between age groups

Lower emotional and cognitive functioning in AYA survivors was observed in comparison with OAs and the elderly, which was also statistically significant after correcting for time since diagnosis, comorbidity and whether patients had received chemotherapy (Table 2). Physical and role functioning was significantly worst for elderly survivors after correction. AYAs and OAs scored significantly higher on financial difficulties compared with elderly survivors.

**Table 2:** Mean EORTC QLQ-C30 scores of sarcoma survivors according to age groups

Mean (±SD)^a^	AYA *N* = 186	OA *N* = 748	Elderly *N* = 165	ANOVA	ANCOVA^b^
Physical functioning	87.8 (17.1)	82.1 (20.5)	73.7 (22.9)	**<0.001**^c^^,^^d^^,^^e^	**<0.001**^d^^,^^e^
Role functioning	83.7 (21.8)	79.0 (26.8)	74.4 (29.7)	**0.006**^d^	0.324
Emotional functioning	80.3 (24.1)	86.1 (19.3)	87.7 (16.5)	**0.001**^c^^,^^d^	**<0.001**^c^^,^^d^^,^^e^
Cognitive functioning	83.6 (22.6)	86.9 (19.5)	87.3 (17.5)	0.123	**<0.001**^c^^,^^d^
Social functioning	85.5 (21.3)	86.6 (21.6)	87.7 (20.7)	0.640	0.055
Global health status/QoL	80.3 (16.4)	78.3 (18.0)	74.1 (19.0)	**0.006**^d^^,^^e^	0.521
Fatigue	22.6 (23.8)	22.4 (23.9)	24.3 (22.8)	0.670	0.126
Nausea/vomiting	2.3 (8.0)	2.8 (9.1)	2.6 (9.1)	0.776	0.581
Pain	15.2 (22.9)	17.9 (25.3)	16.7 (24.0)	0.409	0.034
Dyspnoea	7.0 (16.9)	11.0 (21.2)	16.9 (27.1)	**<0.001**^d^^,^^e^	0.117
Insomnia	15.3 (24.9)	20.2 (28.8)	20.1 (27.3)	0.110	0.384
Appetite loss	4.4 (16.3)	4.8 (15.7)	7.8 (19.4)	0.095	0.455
Constipation	4.4 (12.8)	8.6 (19.9)	11.5 (22.7)	**0.003**^c^^,^^d^	0.454
Diarrhoea	3.2 (10.5)	5.5 (15.7)	4.5 (16.3)	0.184	0.209
Financial difficulties	11.9 (23.7)	9.8 (23.1)	2.4 (9.4)	**<0.001**^d^^,^^e^	**<0.001**^d^^,^^e^

The values in bold indicate a statistically significant *P*-value.

ANCOVA, analysis of covariance; ANOVA, analysis of variance; AYA, adolescents and young adults; OA, older adults; QoL, quality of life.

a Higher score on the functional scales and global QOL indicates better functioning and health-related QoL (HRQoL), whereas a higher score on the symptom scales indicates more complaints.

b Analysis of covariance, corrected for chemotherapy (yes/no), time since diagnosis and comorbidity.

c,d,e Corresponds to statistically significant Bonferroni *post hoc* analysis (*P* < 0.05) for ^c^AYA versus OA, for ^d^AYA versus elderly and for ^e^OA versus elderly.

### HRQoL scores stratified by histological subtype

In Table 3, the scores for several functioning and symptom scales are depicted stratified by age group and by histological subtype. Especially malignant peripheral nerve sheath tumour, chordoma and osteosarcoma showed large clinically relevant differences between survivors and the norm population.

**Table 3:** All functioning scales, global quality of life and two symptom scores (pain and fatigue) stratified by histological subtype and age group. Groups with less than three patients are not reported

	Age group	*N*	PF	RF	EF	CF	SF	QoL	Fatigue	Pain
Norm population	AYA	186	94.1	90.9	86.2	93.9	94	79.2	18.6	12
	OA	901	88.7	87	88.5	91.7	93.4	77.4	17.2	16.6
	Elderly	232	82.6	83.2	88.5	87.2	91.7	76	20.2	18.7
Ewing sarcoma	AYA	16	94.2	89.6	94.3	88.5	90.6	83.3	13.2	11.5
	OA	12	66.7	66.7	79.9	66.7	79.2	73.6	45.4	25.0
	Elderly	0								
Chondrosarcoma	AYA	24	79.4	72.9	72.6	77.8	72.9	72.6	31.0	18.8
	OA	86	78.1	73.5	88.8	87.3	86.5	78.8	20.0	19.2
	Elderly	15	71.1	62.2	87.8	90.0	90.0	76.7	22.2	24.4
Osteosarcoma	AYA	29	76.1	69.5	78.4	80.5	82.8	73.9	32.6	23.0
	OA	34	71.6	69.6	83.1	82.8	78.9	77.0	30.7	23.5
	Elderly	6	47.8	41.7	94.4	88.9	88.9	68.1	29.6	19.4
Chordoma	AYA	1								
	OA	22	68.5	64.4	78.8	77.3	72.0	64.0	38.1	35.6
	Elderly	6	48.8	47.2	83.3	88.9	77.8	58.3	38.0	66.7
Dermatofibrosarcoma protuberans	AYA	24	97.5	88.9	84.4	87.5	94.4	89.2	10.6	12.5
	OA	44	91.7	89.8	89.8	89.4	91.3	88.1	14.4	8.3
	Elderly	3	82.2	66.7	75	83.3	88.9	63.9	33.3	27.8
Leiomyosarcoma	AYA	7	99.0	95.2	60.7	71.4	73.8	79.8	31.7	4.8
	OA	84	86.1	86.3	87.0	87.6	92.2	81.4	17.9	13.7
	Elderly	16	81.7	90.0	87.8	85.6	95.6	79.7	21.5	8.9
Rhabdomyosarcoma	AYA	6	91.1	97.2	80.6	83.3	91.7	83.3	16.7	2.8
	OA	8	80.8	77.1	87.5	89.6	91.7	82.3	16.7	12.5
	Elderly	0								
Synovial sarcoma	AYA	10	86.7	88.3	80.8	83.3	91.7	85.0	18.9	11.7
	OA	23	83.5	71.7	84.1	87.0	82.6	77.5	26.6	19.6
	Elderly	1								
Liposarcoma	AYA	22	94.2	87.9	80.3	87.1	88.6	81.1	20.2	13.6
	OA	125	86.0	82.1	85.4	88.9	85.3	78	22.3	14.9
	Elderly	22	64.2	75	84.2	88.6	87.1	71.2	32.8	14.4
Myxofibrosarcoma	AYA	4	81.7	91.7	83.3	91.7	87.5	77.1	22.2	8.3
	OA	95	85.3	82.1	87.1	89.9	88.5	79.5	17.8	17.2
	Elderly	34	74.6	77.6	87.4	81.4	85.2	73.8	23.3	15.7
Vascular sarcoma	AYA	2								
	OA	29	77.0	78.7	80.5	86.2	85.6	79.0	26.8	17.8
	Elderly	11	81.2	70.0	85.0	88.3	83.3	81.7	21.1	11.7
Malignant peripheral nerve sheath tumour	AYA	9	77.0	85.1	78.7	72.2	87.0	85.2	29.6	18.5
	OA	20	75.7	70.8	85.8	85.0	84.2	79.2	26.7	19.2
	Elderly	3	73.3	61.1	66.7	88.9	77.8	77.8	40.7	22.2

The shades of grey correspond to clinically relevant difference between scores of survivors and the norm population: (white) trivial difference, (light grey) small difference, (dark grey) medium difference, (black) large difference.

AYA, adolescents and young adults; CF, cognitive functioning; EF, emotional functioning; OA, older adults; QoL, global quality of life; PF, physical functioning; RF, role functioning; SF, social functioning.

### Patients, tumour and treatment characteristics associated with low HRQoL scores

Extremity localisation had an odds ratio of 2.35 (95% confidence interval 1.25-4.39) for low physical functioning in AYAs and not in OAs and elderly survivors (Table 4). Chemotherapy as part of the treatment strategy, having a BS and having multiple comorbidities were most often associated with low scores on subscales. A shorter time since diagnosis did not seem to be associated with impaired HRQoL.

**Table 4:** Univariate age-stratified logistic regression analyses amongst sarcoma survivors for the odds of (1) having a clinically relevant worse score on the concerning subscale compared with the normative population versus (0) having no clinically relevant worse score compared with the normative population. The threshold used was for a small clinical importance as determined by Cocks et al.^26^ (2011) in Evidence-Based Guidelines for Determination of Sample Size and Interpretation of EORTC QLQ-C30

Adolescents and young adults (*N* = 186)	Low physical functioning			Low role functioning			Low cognitive functioning			Low emotional functioning		
	OR	95 % CI	*P*	OR	95 % CI	*P*	OR	95 % CI	*P*	OR	95 % CI	*P*
Male	0.93	0.50-1.73	0.82	1.40	0.77-2.55	0.27	1.04	0.57-1.88	0.90	0.80	0.41-1.55	0.50
Chemotherapy	**2.86**	**1.50-5.47**	**<0.01**	**2.24**	**1.19-4.22**	**0.01**	1.69	0.90-3.16	0.10	0.68	0.33-1.39	0.29
Bone sarcoma	**3.09**	**1.63-5.84**	**<0.01**	**3.86**	**2.05-7.27**	**<0.01**	**2.10**	**1.14-3.85**	**0.02**	1.32	0.68-2.56	0.42
<5 Years since diagnosis	0.96	0.51-1.81	0.91	1.50	0.81-2.76	0.20	1.67	0.90-3.08	0.10	1.67	0.85-3.26	0.13
Comorbidities	1.30	0.87-1.95	0.20	**1.58**	**1.04-2.40**	**0.03**	**2.17**	**1.37-3.43**	**<0.01**	1.49	0.97-2.27	0.07
Extremities	**2.35**	**1.25-4.39**	**0.01**	1.17	0.65-2.12	0.60	1.14	0.63-2.06	0.67	1.05	0.54-2.04	0.88

	Low social functioning			Low global QoL			High fatigue			High pain		

Male	1.36	0.74-2.48	0.32	0.90	0.49-1.66	0.74	0.98	0.51-1.86	0.94	1.04	0.53-2.05	0.90
Chemotherapy	**1.99**	**1.06-3.74**	**0.03**	1.49	0.79-2.81	0.22	1.84	0.95-3.56	0.07	1.30	0.65-2.61	0.46
Bone sarcoma	**2.23**	**1.21-4.12**	**0.01**	1.78	0.96-3.30	0.07	1.78	0.93-3.40	0.08	1.75	0.89-3.44	0.11
<5 Years since diagnosis	1.85	1.00-3.43	0.051	1.05	0.56-1.97	0.87	1.47	0.77-2.82	0.25	1.06	0.53-2.12	0.86
Comorbidities	**1.68**	**1.11-2.55**	**0.02**	**2.42**	**1.53-3.82**	**<0.01**	**1.78**	**1.16-2.72**	**<0.01**	**1.55**	**1.01-2.39**	**0.04**
Extremities	1.52	0.83-2.77	0.18	1.23	0.67-2.27	0.50	1.02	0.53-1.93	0.96	1.40	0.71-2.74	0.33
Older adults (*N* = 748)	Low physical functioning			Low role functioning			Low cognitive functioning			Low emotional functioning		

Male	**0.73**	**0.54-0.99**	**0.04**	0.96	0.71-1.29	0.78	**0.74**	**0.55-0.996**	**0.047**	**0.64**	**0.46-0.89**	**<0.01**
Chemotherapy	**2.26**	**1.50-3.39**	**<0.01**	**1.78**	**1.19-2.67**	**<0.01**	**2.04**	**1.36-3.07**	**<0.01**	1.52	0.99-2.34	0.06
Bone sarcoma	1.11	0.56-2.20	0.76	**2.09**	**1.47-2.98**	**<0.01**	1.31	0.92-1.87	0.13	0.88	0.41-1.91	0.75
<5 Years since diagnosis	1.05	0.78-1.41	0.77	1.10	0.82-1.49	0.53	0.85	0.63-1.14	0.29	**0.70**	**0.50-0.98**	**0.04**
Comorbidities	**1.67**	**1.47-1.89**	**<0.01**	**1.49**	**1.32-1.68**	**<0.01**	**1.50**	**1.33-1.70**	**<0.01**	**1.44**	**1.27-1.63**	**<0.01**
Extremities	1.14	0.85-1.53	0.39	0.82	0.61-1.11	0.19	**0.70**	**0.52-0.94**	**0.02**	0.83	0.59-1.15	0.26
	Low social functioning			Low global QoL			High fatigue			High pain		

Male	0.82	0.60-1.10	0.19	0.74	0.54-1.02	0.06	**0.63**	**0.45-0.88**	**<0.01**	**0.63**	**0.46-0.86**	**<0.01**
Chemotherapy	**1.89**	**1.26-2.84**	**<0.01**	1.23	0.80-1.89	0.34	**3.17**	**1.98-5.08**	**<0.01**	1.41	0.93-2.14	0.10
Bone sarcoma	**1.56**	**1.09-2.22**	**0.02**	1.29	0.89-1.88	0.18	**1.57**	**1.06-2.33**	**0.02**	**1.85**	**1.29-2.64**	**<0.01**
<5 Years since diagnosis	0.92	0.68-1.24	0.57	0.78	0.57-1.07	0.12	0.98	0.70-1.36	0.89	0.93	0.68-1.26	0.62
Comorbidities	**1.35**	**1.20-1.52**	**<0.01**	**1.68**	**1.48-1.91**	**<0.01**	**1.75**	**1.52-2.01**	**<0.01**	**1.61**	**1.42-1.83**	**<0.01**
Extremities	**0.70**	**0.52-0.96**	**0.02**	**0.70**	**0.51-0.97**	**0.03**	**0.58**	**0.42-0.82**	**<0.01**	0.87	0.64-1.19	0.39
Elderly (*N* = 165)	Low physical functioning			Low role functioning			Low cognitive functioning			Low emotional functioning		

Male	0.74	0.38-1.43	0.37	0.85	0.43-1.67	0.64	1.12	0.57-2.18	0.75	0.71	0.34-1.50	0.37
Chemotherapy	1.01	0.24-4.20	0.99	0.97	0.21-4.50	0.97	0.19	0.02-1.62	0.13	0.47	0.05-3.99	0.49
Bone sarcoma	**3.55**	**1.40-8.97**	**<0.01**	**6.16**	**2.32-16.4**	**<0.01**	0.80	0.35-1.87	0.61	0.79	0.29-2.12	0.64
<5 Years since diagnosis	0.92	0.48-1.75	0.79	1.04	0.54-2.00	0.91	0.83	0.43-1.60	0.59	1.12	0.53-2.33	0.77
Comorbidities	**1.41**	**1.12-1.76**	**<0.01**	1.22	0.98-1.51	0.07	1.16	0.94-1.43	0.16	**1.36**	**1.08-1.71**	**<0.01**
Extremities	1.02	0.55-1.92	0.94	0.86	0.45-1.63	0.64	0.81	0.43-1.52	0.50	0.63	0.30-1.32	0.22
	Low social functioning			Low global QoL			High fatigue			High pain		

Male	1.06	0.53-2.13	0.67	0.74	0.37-1.46	0.38	0.54	0.27-1.06	0.07	0.81	0.39-1.67	0.57
Chemotherapy	2.61	0.56-12.1	0.22	1.03	0.24-4.50	0.97	0.22	0.03-1.87	0.17	1.88	0.40-8.74	0.42
Bone sarcoma	1.64	0.70-3.81	0.25	1.23	0.53-2.86	0.64	1.14	0.49-2.63	0.76	**2.47**	**1.04-5.87**	**0.04**
<5 Years since diagnosis	0.94	0.47-1.85	0.85	1.65	0.85-3.22	0.14	1.09	0.56-2.11	0.80	1.31	0.65-2.67	0.45
Comorbidities	1.17	0.94-1.44	0.16	**1.35**	**1.08-1.67**	**<0.01**	**1.50**	**1.19-1.91**	**<0.01**	**1.28**	**1.03-1.60**	**0.03**
Extremities	0.92	0.47-1.79	0.80	0.85	0.44-1.63	0.62	0.78	0.41-1.49	0.45	1.03	0.52-2.07	0.93

The demographic and clinical factors that were statistically significant in the univariate logistic regression analysis were then put into a multivariate logistic regression analysis. If the odds ratio remained statistically significant in the multivariate regression analysis, the cells of the table displaying this odds ratio is grey.

## Discussion

This population-based, cross-sectional study assessing HRQoL amongst sarcoma survivors showed that AYA and OA survivors reported statistically significant and clinically meaningful worse physical, role, emotional, cognitive and social functioning in comparison with the normative population, which was not the case for elderly survivors. This suggests that being diagnosed and treated for sarcoma has a greater impact on the functional status of younger survivors than older survivors. A possible explanation for the lesser impact of sarcoma on elderly survivors could be that due to the natural course of aging, a decline in functional status occurs also in elderly without cancer and therefore the difference in HRQoL is not significant. Explanations for the greater impact on younger survivors might be that they have higher work-related and social demands, are emotionally more vulnerable as they are still establishing an identity and lack effective coping strategies since they have never experienced severe illness.

Interestingly, AYA survivors reported significantly worse scores on the functional scales compared with their norm, however they did not report worse scores on the symptom scales. The same phenomenon was seen in AYA patients with thyroid cancer.^27^ A possible explanation might be that younger survivors have more physical resilience than older patients and thus experience symptoms to a lesser extent.^28^ Notably, AYAs received chemotherapy most often and were most often diagnosed with BS in comparison with OAs and elderly. This might play a part in the low functioning scores in AYA survivors, but apparently had no effect on symptom scores.

AYA survivors reported significantly worse scores on emotional and cognitive functioning compared with OA and elderly survivors and worse social functioning compared with elderly survivors. Considering the generally assumed better HRQoL in the young, these striking results emphasise the impact of sarcoma on HRQoL in AYA survivors once again. The time in their lives at which AYA patients are diagnosed is an important time for establishing an identity and making significant life decisions.

Concerning specific sarcoma subtypes, the difference in HRQoL between the normative population and chordoma survivors was striking and once again emphasises the severity of this sarcoma subtype, that is mainly localised in the axial skeleton.^29^ With these small numbers, however, caution has to be taken with generalisations.

Interestingly, survivors who were >5 years since diagnosis did not have significantly better HRQoL scores in comparison with survivors who were <5 years since diagnosis. This suggests that any improvement in HRQoL occurs in the first years after diagnosis and after 5 years no further improvement can be expected in general. A longitudinal investigation of HRQoL in AYA survivors in the first 2 years after diagnosis found that the most improvement in HRQoL occurs within the first year after diagnosis. Improvement in HRQoL was also seen in the second year after diagnosis, although this was far less than in the first year.^30^

The results from this study are in line with studies on HRQoL in thyroid cancer survivors and diffuse large B-cell lymphoma survivors, which both reported that the difference between survivors and the norm population was largest for the younger age group.^27^^,^^31^ A similar study on HRQoL in multiple myeloma survivors did not find age-related differences.^32^ A pooled analysis on the effects of age on HRQoL in cancer populations concluded that HRQoL is generally impaired in cancer patients, however the impact on specific domains varies with age. Young cancer patients had more financial problems and worse social and role functioning in comparison with the general population, which is largely in line with the results from this study.^33^

Limitations of this study include a possible selection bias, since it is unknown whether survivors did not participate due to either a poor health or an absence of symptoms.^34^ A challenge in interpreting these age-stratified results remains the extreme heterogeneity of sarcoma across the age spectrum. This study attempted to take the heterogeneity of subtypes and treatment into account by combining HRQoL outcomes with clinical data. Furthermore, survivorship bias might impede the generalisation of these results to all sarcoma patients. Strengths of this study are that it is population-based and includes a large number of patients. Considering the limited amount of data available on HRQoL in sarcoma survivors, this study is a valuable addition and provides more insight into the impact of sarcoma on HRQoL and the role of age.

In order to meet the needs of sarcoma survivors on physical, emotional, role, cognitive and social functioning, a specific measurement strategy for HRQoL in the extremely heterogenous sarcoma survivor population is essential.^35^ The incorporation of age-specific questions is crucial in such a measurement strategy to meet the age-specific needs of sarcoma survivors. The benefits of addressing the psychological and social issues in sarcoma patients may be lifelong, especially in younger patients who are establishing an identity and making important life choices that will affect the rest of their lives. The results from this study emphasise the need for the development of appropriate psychological and social interventions. Ideally, the psychological and social needs and issues in sarcoma patients should at least always be addressed at some point throughout the disease trajectory.

## Conclusion

In conclusion, this nationwide study shows that in a very heterogeneous sarcoma survivor population, there is also a wide variety in HRQoL scores. Nevertheless, clear age-related patterns in HRQoL are observed showing sarcoma has a greater impact on younger survivors. These results emphasise the need for personalised follow-up care that not only includes risk-adjusted care related to disease relapse, but also age-adjusted care to address the needs that impact important aspects of HRQoL.
